# Functional Analysis of BcBem1 and Its Interaction Partners in *Botrytis cinerea*: Impact on Differentiation and Virulence

**DOI:** 10.1371/journal.pone.0095172

**Published:** 2014-05-05

**Authors:** Sabine Giesbert, Ulrike Siegmund, Julia Schumacher, Leonie Kokkelink, Paul Tudzynski

**Affiliations:** Institut für Biologie und Biotechnologie der Pflanzen, Westfälische Wilhelms Universität, Münster, Germany; Zhejiang University, China

## Abstract

In phytopathogenic fungi the establishment and maintenance of polarity is not only essential for vegetative growth and differentiation, but also for penetration and colonization of host tissues. We investigated orthologs of members of the yeast polarity complex in the grey mould fungus *Botrytis cinerea*: the scaffold proteins Bem1 and Far1, the GEF (guanine nucleotide exchange factor) Cdc24, and the formin Bni1 (named Sep1 in *B. cinerea*). BcBem1 does not play an important role in regular hyphal growth, but has significant impact on spore formation and germination, on the establishment of conidial anastomosis tubes (CATs) and on virulence. As in other fungi, BcBem1 interacts with the GEF BcCdc24 and the formin BcSep1, indicating that in *B. cinerea* the apical complex has a similar structure as in yeast. A functional analysis of BcCdc24 suggests that it is essential for growth, since it was not possible to obtain homokaryotic deletion mutants. Heterokaryons of Δcdc24 (supposed to exhibit reduced *bccdc24* transcript levels) already show a strong phenotype: an inability to penetrate the host tissue, a significantly reduced growth rate and malformation of conidia, which tend to burst as observed for Δ*bcbem1*. Also the formin BcSep1 has significant impact on hyphal growth and development, whereas the role of the putative ortholog of the yeast scaffold protein Far1 remains open: Δ*bcfar1* mutants have no obvious phenotypes.

## Introduction

The grey mould fungus *Botrytis cinerea* is a broad host range plant pathogen that is able to infect different tissues of more than 200 plants, causing serious crop losses worldwide [1]. Its economic importance, its easy experimental accessibility and the availability of a broad set of molecular tools has made it an interesting model system for a necrotrophic pathogen [2]. In recent years, many signaling factors have been identified that are involved in disease development, including mitogen-activated protein kinases (MAPK), components of the calcium cascade, the cAMP pathway and reactive oxygen species (ROS)/redox signaling systems [3]. The ability to form polarized cells is of major importance for fungi in general and especially for pathogens; accordingly in *Botrytis* polarity generation is important for mycelial growth, for many developmental stages (conidia, sclerotia, fruiting bodies) and especially for penetration and colonization of the host tissue. The molecular basis for establishment and maintenance of polarity has been intensively studied in yeast and model filamentous fungi such as *Neurospora crassa, Aspergillus nidulans* and *Ustilago maydis* [4]–[6]; it requires the concerted action of several protein complexes at the hyphal tip, the so-called polarisome, the Spitzenkörper and the exocyst [7], [8]. In yeast the polarity complex includes several signaling components, amongst others monomeric GTPases such as Cdc42p, guanine nucleotide exchange factors (GEFs) such as Cdc24p, p21-activated kinases (PAKs) such as Ste20p and Cla4p, the formins Bni1p and Bnr1p establishing the link to the actin cytoskeleton, and the scaffold proteins Bem1p and Far1p (linking the Gβy dimer of heterotrimeric G proteins with Cdc24p and Bem1p) [9]–[12]. In filamentous fungi there are additional polarity-determining components such as the GTPase Rac and the NADPH oxidase complex [6], [13]. In addition, the conserved components may have different importance and functions, e.g. in contrast to yeast Δ*cdc42* mutants of filamentous fungi are not lethal and not even severely impaired in polar growth, as was recently also shown in *B. cinerea* [14]. This holds also true for the central scaffold protein Bem1, which in filamentous fungi - in contrast to yeast - does not seem to be essential but has varying impact on hyphal growth [15]–[17]. However, it plays important (and varying) roles in several polarity requiring fungal processes like oriented hyphal growth *in planta* (*Epichloe festucae*), conidia formation (*N. crassa* and *A. nidulans*), germination (*N. crassa*) and the fusion of conidial anastomosis tubes (CATs) in *N. crassa* and *E. festucae* [17], [18]. Here we show that the *B. cinerea* Bem1 ortholog (BcBem1) has even less effect on normal hyphal growth than in *N. crassa*, where Δ*bem-1* mutants exhibit slightly reduced growth rates, in *B. cinerea* it is important for the formation and germination of conidia and for proper penetration and colonization of the host tissue. We confirm that - in spite of obviously not being essential for growth and polarity, also in this system Bem1 interacts with the ortholog of a putative complex partner, Cdc24, and we present for the first time functional analyses data for the *B. cinerea* orthologs of the yeast Bem1 complex partners Cdc24p, Far1p, and Bni1p.

## Results

### Identification and deletion of the *bem1* ortholog in *B. cinerea* B05.10

The ortholog of *bem1* in *B. cinerea* (*bcbem1*) was identified in the *B. cinerea* database (Broad Institute) due to its similarity with Bem1p from *S. cerevisiae*. The annotation was confirmed by cDNA sequencing and results in an open reading frame (ORF) of 2,010 bp with two introns of 74 bp and 49 bp, encoding for a protein of 628 aa. BcBem1 shows 32%, 55%, 60% and 63% to the characterized orthologs of *S. cerevisiae* (Bem1p), *A. nidulans* (BemA), *E. festucae* (BemA) and *N. crassa* (Bem-1) and contains the typical Bem1 features: two SH3 (Src homology 3; IPR001452) domains that may mediate protein-protein interactions and binding to Cdc42, one PX (phox homologous; IPR001683) domain known to mediate phosphoinositide binding and one PB1 (Phox/Bem1p; IPR000270) domain that has been shown to interact with the PB1 domain of Cdc24 in yeast [19] (Fig. S1A).

In order to functionally characterize BcBem1, deletion mutants of *bcbem1* (Δ*bcbem1*) were generated via replacement of the ORF by a hygromycin resistance cassette (*hph*) in *B. cinerea* strain B05.10 (wild type, WT) (Fig. S1B). Diagnostic PCR and Southern blot analysis revealed the absence of *bcbem1* and furthermore the absence of additional ectopic integrations of the replacement construct in three independent mutants. (Fig. S1C). As the mutants showed similar phenotypes in all experiments; the data for one arbitrarily chosen mutant (Δ*bcbem1*-18) are presented. Complementation of this deletion mutant (Δ*bcbem1*C) was accomplished by re-introduction of *bcbem1* under control of its native promoter (for details, see Materials and Methods, Table 1, Table S1).

**Table 1:** *B. cinerea* strains used in this study.

Strain	Genotype	Reference
WT:B05.10	Isolate from *Vitis vinifera*, *MAT1-1*	[67]
Δ*bcbem1*	B05.10, Δ*bcbem1::hph*, homokaryon	This study
Δ*bcbem1*C	B05.10, Δ*bcbem1::hph*, *bcbem1::nat1*, heterokaryon	This study
GFP-BcBem1	B05.10, Δ*bcbem1::hph*, P*oliC::gfp-bcbem1::nat1*, heterokaryon	This study
Δ*bccdc24*-het	B05.10, Δ*bccdc24::hph*, heterokaryon	This study
PA3135	B05.10, T-DNA::*hph*, ∼1.0 kb upstream of *bcsep1*, heterokaryon	[32]
Δ*bcsep1*	B05.10, Δ*bcsep1::hph*, homokaryon	This study
Δ*bcfar1*	B05.10, Δ*bcfar1::hph*, homokaryon	This study

### Deletion of *bcbem1* affects the formation of reproductive structures

**Figure 1:**
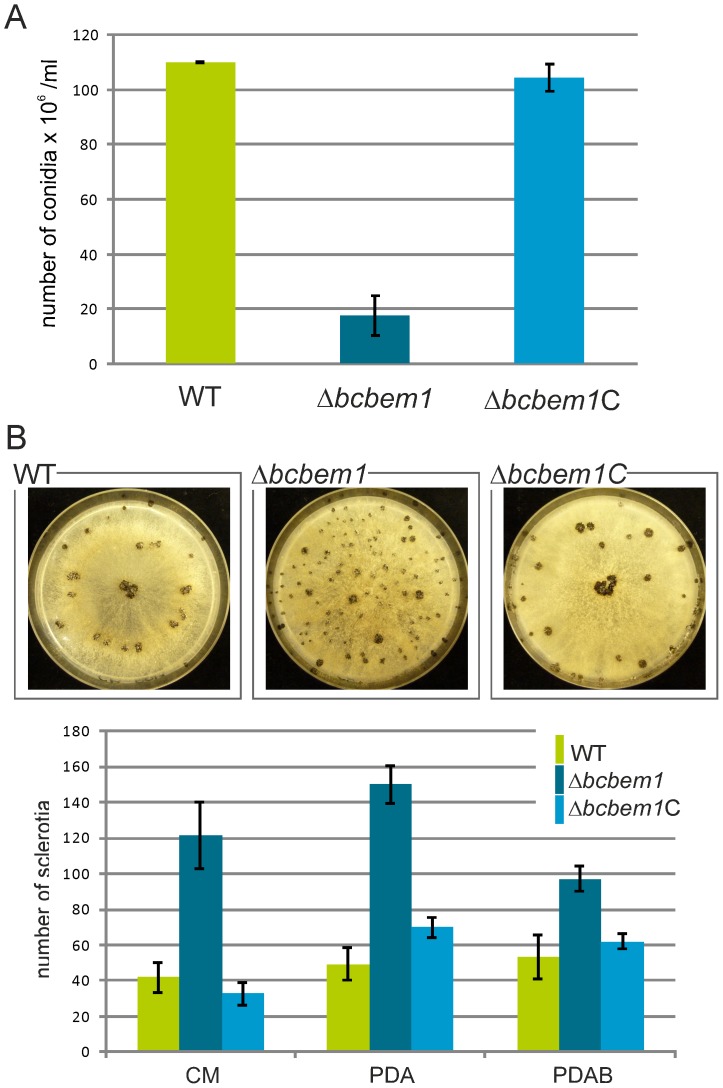
Deletion of *bcbem1* affects the production of conidia and sclerotia. (**A**) Δ*bcbem1* is severely impaired in conidiation. Wild type (WT), Δ*bcbem1* and the complemented strain Δ*bcbem1C* were incubated for 14 d on solid complete medium. Then, conidia were collected and quantified. (**B**) Δ*bcbem1* produces more sclerotia on different rich media. Sclerotia production was tested on different solid media: synthetic complete medium (CM), potato dextrose agar without (PDA) and PDA with 10% mashed bean leaves (PDAB). Cultures (10 per strain and condition) were incubated for 14 d in constant darkness. Sclerotia formed by wild type B05.10, Δ*bcbem1* and Δ*bcbem1*C on CM (upper panel); numbers of sclerotia produced on different media (lower panel).

**Figure 2:**
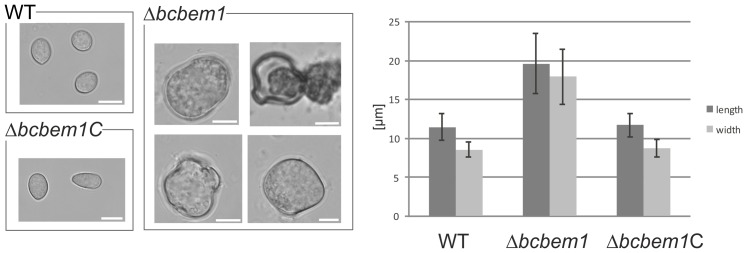
Conidia of Δ*bcbem1* are enlarged and malformed. Conidia of wild type, Δ*bcbem1* and the complemented strain Δ*bcbem1C* were collected from 7-d-old cultures. Widths and lengths of conidia were measured. Scale bars  = 10 µm.

**Figure 3:**
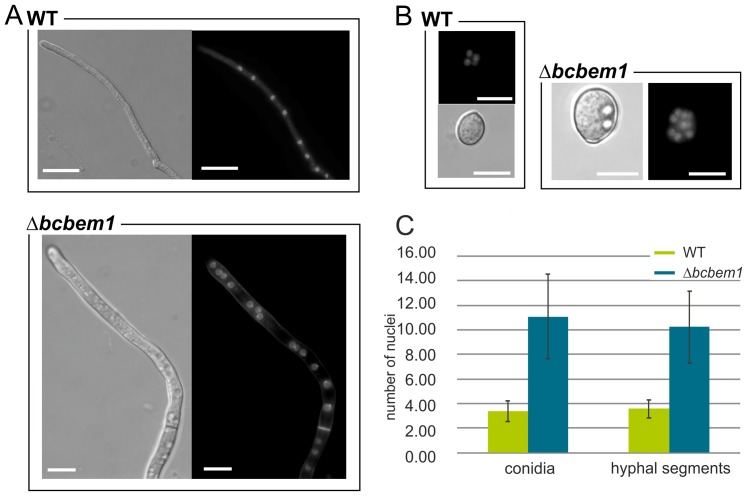
BcBem1 has impact on nuclear contents of conidia and hyphae. Growing hyphae (**A**) (conidia incubated for 16 h in GB5 +2% glucose medium) and ungerminated conidia (**B**) of the wild type and the Δ*bcbem1* mutant were stained with the nucleic acid-specific dye Hoechst 33342. (**C**) Indicated are the numbers of nuclei per conidium and hyphal segment, respectively (40 each). Segments throughout the hyphae were randomly chosen from independent germlings. Scale bars  = 10 µm.

The comparison of radial growth rates of Δ*bcbem1* mutants with those of the WT on rich (complete medium, CM) and minimal medium (Czapek-Dox, CD and Gamborg B5 with glucose) did not reveal significant differences. In addition, the responses of the mutants to the exposure to oxidative (caused by 10 mM H_2_O_2_ or 500 µM menadione) and osmotic stresses (1 M sorbitol, 0.8 M NaCl) were comparable to the recipient strain B05.10 suggesting that BcBem1 is not involved in regulation of hyphal growth and stress resistance (data not shown). Nevertheless, the deletion of *bcbem1* significantly affected the formation of reproduction structures, i.e. of macroconidia (asexual) and sclerotia (asexual and sexual). During incubation in light that promotes conidiation the deletion mutants formed fewer conidia (17% of wild type) (Fig. 1A), while during incubation in continuous darkness the mutant produced more sclerotia than the wild type on different media (290% of wild type on CM) (Fig. 1B). The few conidia produced by the deletion mutants were malformed: they were significantly enlarged (approx. two-fold of wild-type conidia) and irregular in shape (Fig. 2). Staining of conidia and young hyphae with the nucleic acid-specific dye Hoechst 33342 revealed roughly two-fold increased numbers of nuclei in the conidia and hyphal segments (segments distributed throughout the hyphae were analyzed) of the Δ*bcbem1* mutant (Fig. 3). Septa of WT and Δ*bcbem1* were stained with calcofluor white, revealing regular septation of the hyphae (data not shown). Taken together, BcBem1 is necessary for the differentiation of conidia.

### BcBem1 has impact on conidial germination and related processes

**Figure 4:**
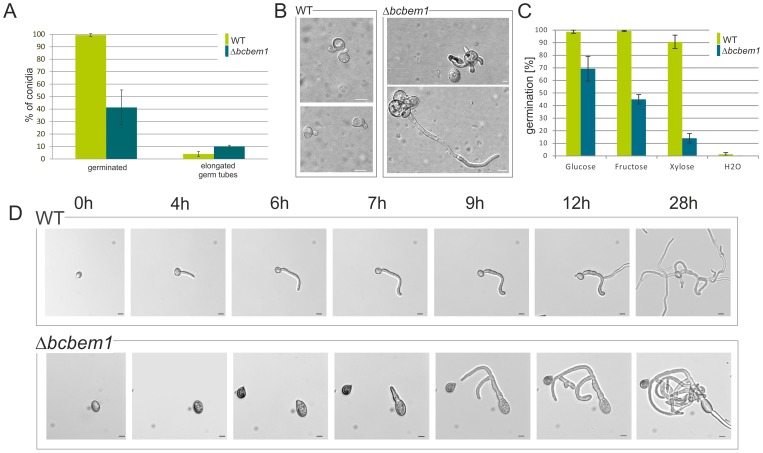
Germination capabilities of Δ*bcbem1* conidia are impaired. Germination rates (**A**) and the germ tube morphology (**B**) are altered on hydrophobic surfaces. Conidial suspensions of strains (1×10^5^/ml in H_2_O) were incubated on polypropylene foil in a humid chamber for 24 h. Total numbers of germinated conidia and conidia forming elongated germ tubes were counted. Experiment was done in triplicates; mean values and standard deviations were calculated from 300 conidia per strain and condition. Scale bars  = 10 µm. (**C**) Nutrient-induced germination rates of Δ*bcbem1* conidia are significantly decreased. Conidia (5×10^5^/ml) were incubated for 24 h in liquid GB5 supplemented with the indicated carbon sources (10.5 M) on glass surfaces. Experiment was done in triplicates; mean values and standard deviations were calculated from 300 conidia per strain and condition. (**D**) Germination kinetics and germ tube morphology of Δ*bcbem1* conidia are different from wild-type conidia. Time course of nutrient-induced germination (in GB5 +2% glucose) is shown. Scale bars  = 10 µm.

To see if the germination ability of the morphologically altered Δ*bcbem1* conidia differs from the wild type various germination assays were conducted. Generally, germination of conidia of *B. cinerea* can be induced by application of nutrients (on hydrophilic surfaces) and by hydrophobic surfaces (in the absence of nutrients) [20]. Characteristically, on polypropylene foil wild-type conidia form “nose-like” structures immediately after germination. In contrast, only 40% of the Δ*bcbem1* conidia germinated, and an increased number of conidia formed prolonged, irregular germ tubes (10% of all conidia) (Fig. 4A), suggesting an alteration in surface sensing caused by the absence of *bcbem1*. Besides, and as shown in Figure 4C, the germination rates of Δ*bcbem1* conidia were significantly reduced on hydrophilic surfaces and differences were observed for the used carbon sources, i.e. glucose, fructose and xylose. With glucose about 70% of the Δ*bcbem1* conidia had germinated after 24 h, while with fructose and xylose only 45% and 15% had germinated, respectively (Fig. 4C), compared to 97–98% of the wild-type conidia. The fate of the Δ*bcbem1* conidia were studied in more detail using glucose as carbon source. Time course experiments revealed an altered mode of germination of the conidia. Hence, germination was delayed and formed germ tubes grew in circles even after branching events had taken place (Fig. 4D); similar results were observed in CM medium (data not shown). In contrast, wild-type germ tubes grew more or less straight and spread through branching; the hyphae curved slightly as well, but growth appeared more directed than in the mutant. After 48 h the hyphal networks of the WT were more planar and evenly spread, while the mutant hyphae grew more nodular and in piles (data not shown). Notably, we observed that Δ*bcbem1* conidia tended to burst (approx. 6% of conidia) (data not shown).

**Figure 5:**
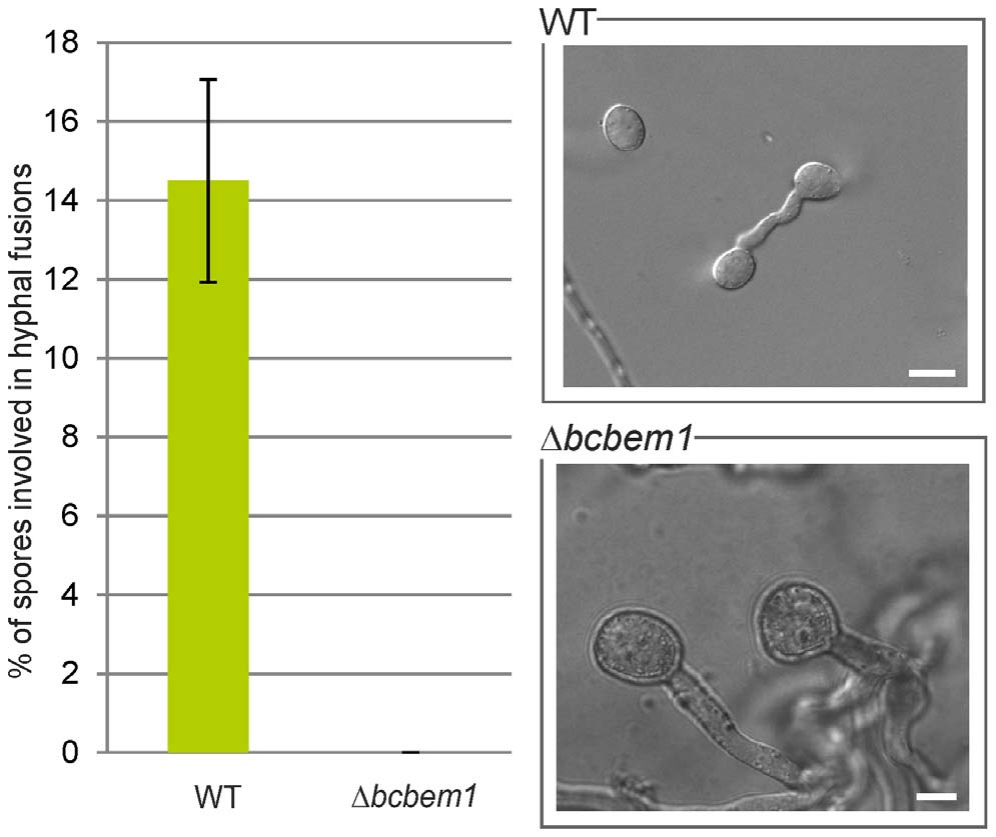
Conidia of Δ*bcbem1* are impaired in CAT (conidial anastomosis tube) formation and fusion. WT- and Δ*bcbem1*-derived conidia (3×10^6^) were incubated for 18 h on Vogel's MM medium. 300 conidia were evaluated for each strain, mean values and standard deviations were calculated, and three replicates yielded similar results. Scale bars  = 10 µm.

Since the Δ*bcbem1* mutant is defective in the two earliest differentiation processes, the formation and germination of conidia, we were interested if the Δ*bcbem1* germlings were able to form conidial anastomosis tube (CAT) fusions. CAT fusions of *B. cinerea* conidia can be induced by germination of conidia (high concentration) on Vogel's minimal medium [21], a process which is disturbed in Δ*bem-1* mutants of *N. crassa* [17]. Interestingly, in contrast to *N. crassa*, where the fusion events are only reduced and delayed, we did not observe any CAT fusions for the Δ*bcbem1* mutant (Fig. 5). Whether this defect is attributed to the mutant's general dysfunction in conidiogenesis and germination, or if CAT fusions themselves are affected by BcBem1 remains open.

### BcBem1 is required for proper penetration and full virulence

**Figure 6:**
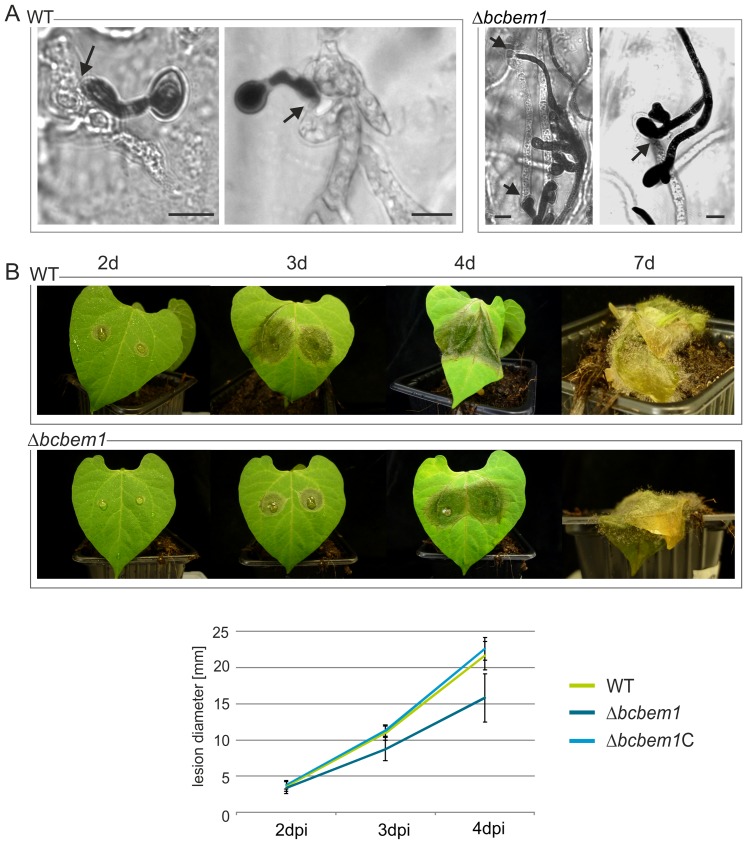
BcBem1 is involved in the infection process. (**A**) Δ*bcbem1* mutants form branched structures before penetrating onion epidermal cells. Hydrophobic sides of heat-inactivated onion epidermis were inoculated with 10-µl droplets of conidial suspensions (5×10^4^/ml in water) and incubated for 24 h at 20°C under humid conditions. Lactophenol blue staining was used to distinguish between fungal hyphae growing on the surface (stained) and inside the epidermal cells (colorless), respectively. Arrows indicate sites of penetration. Scale bars  = 10 µm. (**B**) Δ*bcbem1* is retarded in colonization of French bean (*Phaseolus vulgaris*). Primary leaves of 10-d-old plants were inoculated with 7.5-µl droplets of conidial suspensions (2×10^5^/ml in Gamborg B5 +2% glucose). Statistical evaluation of infection was realized by quantification of lesion diameters at 2, 3 and 4 dpi. Mean values and standard deviations were calculated from 16 lesions (with two measurements) per strain.

The most important differentiation process in pathogenic fungi based on polar growth is the development of functional penetration structures (requiring the re-orientation of growth) and the colonization of the host tissue. The penetration ability of Δ*bcbem1* conidia was studied on heat-inactivated onion epidermal strips. After 24 hours, samples of wild type and the mutant were stained with lactophenol blue to distinguish between hyphae growing on the top of the plant surface (stained) and inside the plant tissue (unstained), respectively. Microscopic analysis revealed that most wild-type conidia formed short germ tubes penetrating the epidermal cells via appressorium-like structures (Fig. 6A, arrows), whereas Δ*bcbem1* conidia formed elongated and branched germ tubes (resembling infection cushions) before penetrating the underlying plant cells (Fig. 6A).

Penetration and subsequent disease progression by Δ*bcbem1* was studied in more detail on primary leaves of young French bean plants (*Phaseolus vulgaris*) using conidial suspensions of wild type and Δ*bcbem1* as inoculum. Two days post inoculation (dpi) the wild type as well as the complemented strain Δ*bcbem1*C had formed primary lesions, visible as brown, necrotic spots. At 7 dpi, both strains had colonized the whole plant and started to produce conidia. The mutant provoked primary lesions after two days as well; however, lesion sizes after three days were significantly smaller than those caused by the wild type (Fig. 6B). Nevertheless, the deletion mutant was able to complete the infection cycle and to produce conidia. In an assay in which primary leaves were inoculated with mycelial plugs of the strains, the Δ*bcbem1* mutant was also delayed in infection (Fig. S2), ruling out that the retarded infection is due to the disturbed germination process of Δ*bcbem1* conidia. In summary, the Δ*bcbem1* mutant is still able to form appressoria-like structures, to penetrate and to colonize the plant tissue, but these processes are significantly delayed.

### BcBem1 localizes in the cytoplasm, the septa and rarely at hyphal tips

**Figure 7:**
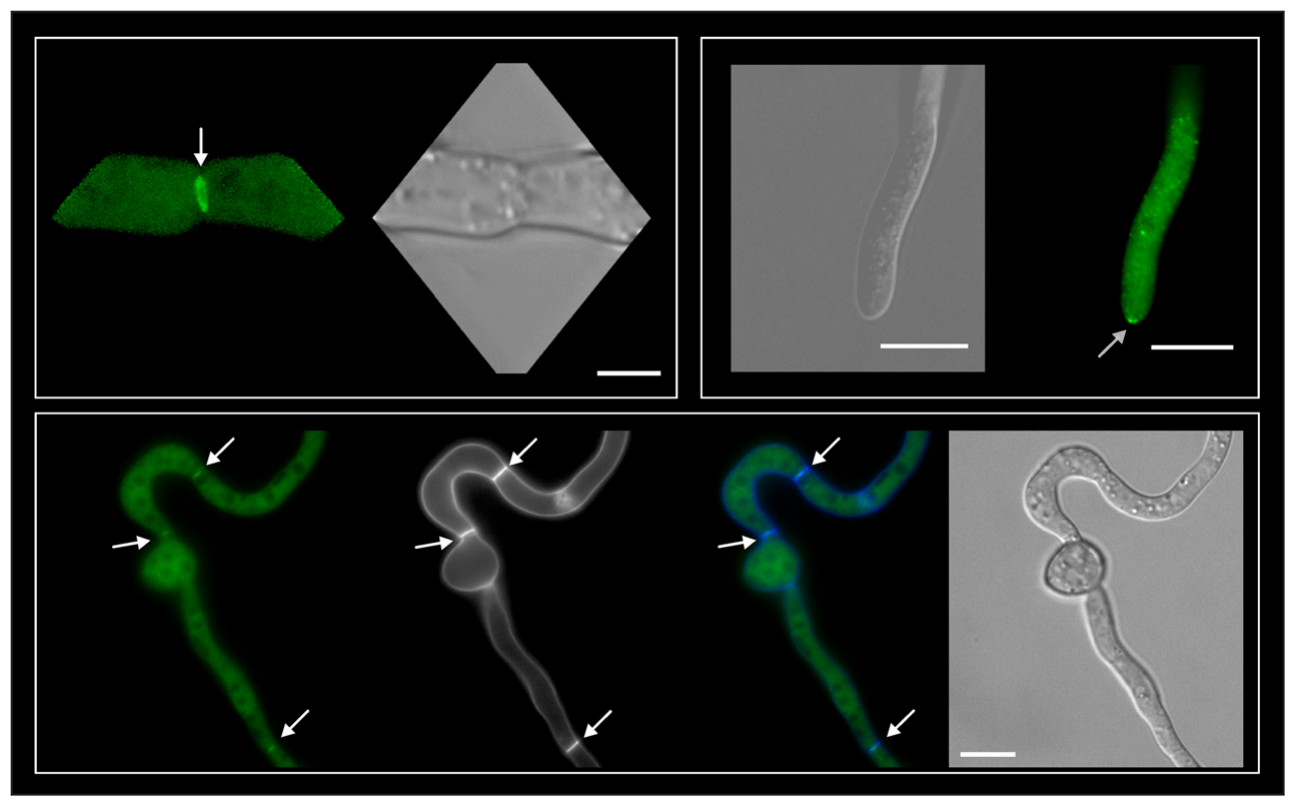
GFP-tagged BcBem1 localizes to the cytoplasm, the septa and the hyphal tips. A *gfp-bcbem1* fusion was expressed from the constitutive *A. nidulans oliC* promoter in the Δ*bcbem1* background. Bottom row shows co- staining of the septae with calcofluor white (CW) (from left to right: GFP, CW, overlay, DIC). For fluorescence microscopy, conidia were incubated for 18 h in GB5 +2% glucose on microscope slides. White arrows indicate septa, grey arrow indicates tip localization. Scale bars  = 10 µm.

To study the subcellular localization of BcBem1, strains were generated expressing N-terminal GFP fusion constructs under control of the constitutive *oliC* promoter from *A. nidulans* in the Δ*bcbem1* background (for more details, see Material and Methods). The GFP-BcBem1 fusion construct was shown to be functional, as it complemented the Δ*bcbem1* phenotype with regard to conidiation, conidia size and virulence on *P. vulgaris* (data not shown). Germlings of these strains were analyzed by epifluorescence microscopy. Surprisingly, in contrast to other fungi, where the Bem1 orthologs were detected mainly at the hyphal tip [16], [17], , localization of BcBem1 was prominently observed at the septa throughout the hyphae, independent of the age of the hyphae. GFP fluorescence and co-staining with calcofluor white shows association of BcBem to the septa. Additionally, BcBem1 localization was visible in the cytoplasm, and only rarely at the hyphal tips (Fig. 7); part of this phenotype (especially the cytoplasmic localization) could be due to the overexpression of the fusion construct.

### BcBem1 is part of a polarity complex involving the GEF BcCdc24

**Figure 8:**
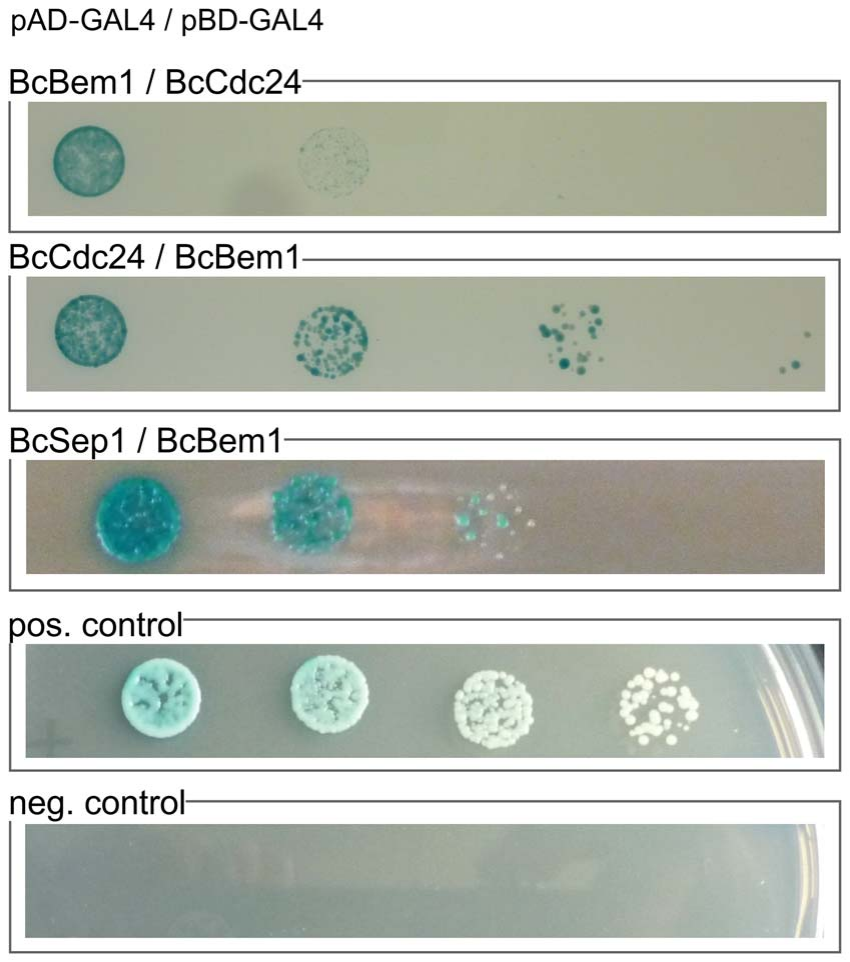
BcBem1 interacts with the GEF BcCdc24 and the formin BcSep1 in a yeast-two-hybrid assay. *S. cerevisiae* strain PJ69-4A was transformed with prey and bait vectors, pAD-GAL4-2.1 and pBD-GAL4-Cam, as indicated and plated on SD-Leu/-Trp/-His/+X-Gal medium to demonstrate the interactions of prey and bait proteins. Positive control: pAD-CpRac^G17V^/pBD-CpCla4 (*Claviceps purpurea*), negative control: empty pBD/pAD. Dropped dilutions of strains were 1, 1∶10, 1∶100, and 1∶1000. Vectors containing BcCdc24, BcBem1 and BcSep1 were transformed with corresponding empty vectors to prove the absence of auto-activation (data not shown). For more details, see Material and Methods.

It was previously shown that Bem1 is part of the polarity complex in yeast, involving also the small GTPase Cdc42, the GEF Cdc24 and the formin Bni1p [9]. Also in several filamentous fungi, e.g. in *A. nidulans* and *E. festucae* a role for Bem1 in the apical complex was suggested [15], [16]. To investigate the composition of the apical complex in *B. cinerea*, direct protein-protein interactions of BcBem1 with suspected interaction partners were tested using the standard yeast two-hybrid (Y2H) system. A search in the *B. cinerea* database identified *bona fide* orthologs of the guanine nucleotide exchange factor Cdc24p (BcCdc24), and the formin Bni1p (BcSep1) from yeast (Table 2); the GTPase BcCdc42 has been described before [14]. cDNA of these genes were cloned into standard prey and/or bait Y2H vectors and transformed into the yeast strain PJ69-4A. His3 and lacZ reporter activities of yeast strains co-expressing BcBem1 and BcCdc24 or BcBem1 and BcSep1 were detected (on SD-leu-trp+X-Gal) demonstrating that BcBem1 interacts with the GEF BcCdc24 and the formin BcSep1 (Fig. 8). In contrast, no growth of yeast strains expressing BcBem1 or BcCdc24 together with the wild-type or the constitutively active version of BcCdc42 were observed (data not shown). However, this does not prove that BcCdc42 does not interact with the scaffold protein BcBem1 and the GEF BcCdc24. Even in systems like e.g. *Claviceps purpurea* where CpBem1 strongly interacts with CpCdc24 and the GEF role of Cdc24 for Cdc42 has been proven, a direct interaction between CpCdc42 and CpBem1 in the yeast-two-hybrid system could not be shown [22]. Still, here we focused on the functional characterization of the verified interaction partners of BcBem1, BcCdc24 and BcSep1.

**Table 2:** Genes/proteins of *B. cinerea* analysed in this study.

		GeneIDs (Broad Institute)		Revised annotation (cDNA sequencing)	
Name	Description	1^st^ annotation	2^nd^ annotation	ORF/introns	Protein
BcBem1	Scaffold protein	BC1G_03145 (628 aa)	B0510_2961 (625 aa)	1,887 bp with two introns (74 bp, 49 bp)	628 aa
BcCdc24	GEF	BC1G_09394 (1,066 aa)	no gene call	3,129 bp with 5 introns (253 bp, 47 bp, 57 bp, 55 bp, 87 bp)	1,042 aa
BcSep1	Formin/Bni1p	BC1G_10712 (1,648 aa)	B0510_3788 (1,766 aa)	5,301 bp with one intron (49 bp)	1,766 aa
BcFar1	Scaffold protein	BC1G_08236 (1,220 aa)	B0510_6879 (1,173 aa)	3,522 bp with four introns (107 bp, 73 bp, 52 bp, 59 bp)	1,173 aa

### BcCdc24 has significant impact on germination, penetration and growth

**Figure 9:**
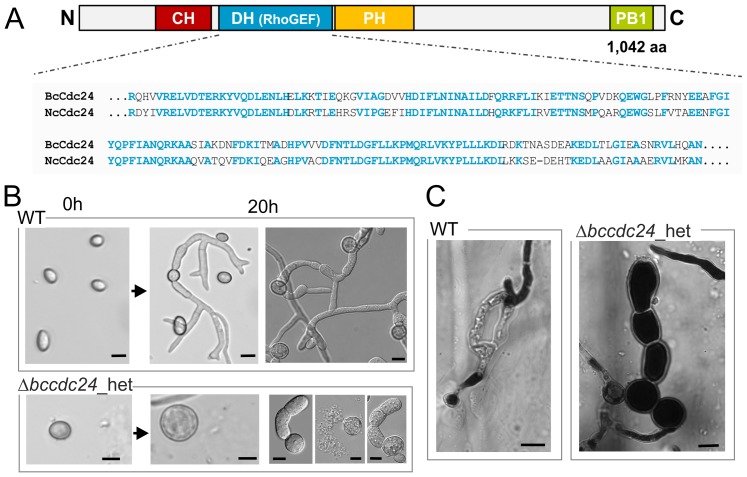
*Bccdc24* is likely an essential gene in *B. cinerea*. (**A**) Domain structure of BcCdc24. CH - calponin homology (IPR001715), DH - Dbl homology/RhoGEF (IPR000219) domain, PH - pleckstrin homology domain (IPR001849), PB1 - Phox/Bem1p domain (IPR000270). Alignment of the DH/RhoGEF domains of Cdc24 protein sequences from *B. cinerea* and *N. crassa* is shown. (**B**) Conidia derived from the Δ*bccdc24* heterokaryon (Δ*bccdc24*_het) supposed to exhibit decreased *bccdc24* expression levels are severely affected in their integrity (tendency to burst) and their capability to germinate on glass surfaces in liquid GB5 +2% glucose medium. (**C**) Δ*bccdc24*_het mutants are impaired in penetration of onion epidermis. Hydrophobic sides of heat-inactivated onion epidermis were inoculated with conidial suspensions and stained with lactophenol blue after 24 h of incubation. Arrows indicate sites of penetration. Scale bars  = 10 µm.

GEFs are proteins involved in the activation of small GTPases by catalyzing the dissociation of GDP allowing the binding of GTP. BcCdc24 possesses the characteristic domains of Rho-GEFs: a DH (Dbl homology; IPR000219) domain mediating GEF activity specific for a number of Rho family members, a PH domain (pleckstrin homology; IPR001849), and a C-terminal PB1 domain (Phox/Bem1p; IPR000270). Moreover, an N-terminal CH (calponin homology; IPR001715) domain was identified that belongs to a superfamily of actin-binding domains found in both cytoskeletal proteins and signal transduction proteins (Fig. 9A).

A replacement approach according to the strategy used for *bcbem1* was initiated to generate *bccdc24* deletion mutants (for details, see Material and Methods). Two hygromycin-resistant transformants that had undergone homologous integration of the Δ*bccdc24* construct without further ectopic integrations were identified by diagnostic PCR and Southern blot analysis (data not shown). However, all attempts to generate homokaryotic strains by isolation of single colonies derived from conidia and protoplasts (four rounds from conidia and three rounds from protoplasts) failed, as *bccdc24* alleles were still detectable by diagnostic PCR (intensities of WT allele were variable but no trend to decrease was observed).

One analyzed heterokaryotic Δ*bccdc24* strains showed a significant growth defect, although it still contained wild-type nuclei (data not shown). As it was previously described that – apart from *N. crassa* [23] – Cdc24 is essential in other filamentous fungi [16], [22], [24], we presumed that Cdc24 might be essential in *B. cinerea* as well and conducted further preliminary analyses with the Δ*bccdc24* heterokaryon (named Δ*bccdc24*_het) supposed to exhibit reduced *bccdc24* transcript levels.

As in other fungi a close association of Bem1 and Cdc24 is described, similar assays were performed with Δ*bccdc24*_het as with Δ*bcbem1*. Besides the significant growth inhibition mentioned above, germination and penetration are severely affected in the heterokaryon: after 20 h conidia of Δ*bccdc24*_het had not yet germinated, but most of them showed a spherical growth which achieved a diameter of up to 22 µm, compared to ∼12 µm of wild-type conidia; some conidia burst and less than 10% germinated but stopped growing afterwards (Fig. 9B). Similar germination behavior was observed on onion epidermis; here the mutant was unable to penetrate after 24 h (Fig. 9C).

### The formin BcSep1 is essential for septum formation and polarized growth

**Figure 10:**
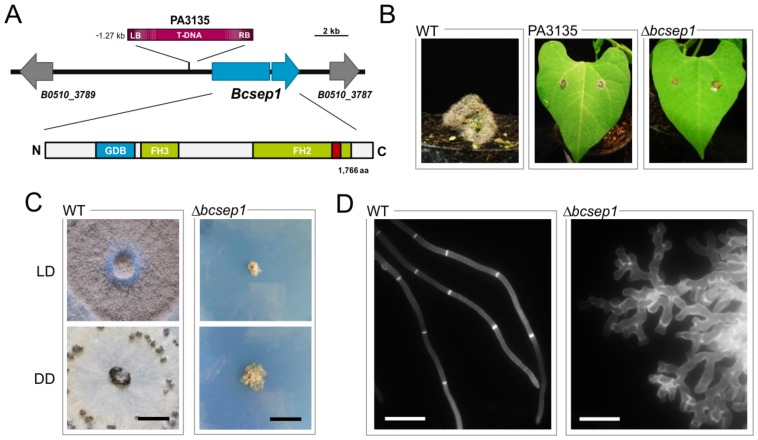
The formin BcSep1 is essential for hyphal growth and virulence. (**A**) *Bcsep1* was identified as virulence-associated gene by a random mutagenesis approach (*Agrobacterium tumefaciens*-mediated transformation). Mutant PA3135 contains a T-DNA insertion upstream of *bcsep1*. The genomic region in strain B05.10 with annotated genes and the domain organization of the encoded protein is shown. GBD - Diaphanous GTPase-binding domain (IPR010473), FH3 - Diaphanous FH3 domain (IPR010472), FH2 - Formin homology 2 domain (IPR015425), DAD (red bar) - diaphanous autoregulatory domain (IPR014767). (**B**) The T-DNA insertion and the deletion of *bcsep1* result in loss of virulence. Primary leaves of *P. vulgaris* were inoculated with mycelial plugs of the wild type, the ATMT mutant PA3135 and the deletion mutant Δ*bcsep1*. Pictures were taken 10 dpi. (**C**) *Bcsep1* deletion mutants form compact undifferentiated colonies. Strains were incubated for 14 d on solid complete medium in light-dark (LD) and continuous darkness (DD), respectively. Scale bars  = 1 cm. (**D**) BcSep1 is required for polarized growth and septum formation. Wild type and Δ*bcsep1* were grown for 2 d and 14 d in liquid complete medium, respectively. Before fluorescence microscopy, hyphae were treated with calcofluor white to stain the cell walls and septa. Scale bars  = 20 µm.

Formins participate in the assembly of the actin and microtubule cytoskeletons in processes like cell division, migration, and development and are regulated by Rho-type GTPases [25]. BcSep1 consists of 1,766 aa and possesses protein domains that are characteristic for diaphanous-related formins (DRF) i.e. an N-terminal GTPase-binding domain (GBD; IPR010473) and a C-terminal diaphanous autoregulatory domain (DAD; IPR014767) as well as formin homology (FH2, FH3; IPR015425, IPR010472) domains (Fig. 10A). The protein sequence shares 34%, 31%, 26%, 60% and 65% aa identity with the characterized orthologs from *S. cerevisiae* (Bni1p; [26]), *Ashbya gossypii* (Bni1; [27]), *Schizosaccharomyces pombe* (Cdc12p; [28]), *A. nidulans* (SepA; [29], [30]) and *N. crassa* (BNI-1; [31]).

In parallel to this study, we identified *bcsep1* as a virulence-associated gene by performing a random mutagenesis approach via *Agrobacterium tumefaciens*-mediated transformation (ATMT) [32]. TAIL-PCR analyses revealed that the left border (LB) of the T-DNA was inserted 1,270 bp upstream of *bcsep1* in the non-pathogenic transformant PA3135. Further PCR analyses were performed but failed to detect the insertion site of the right border (RB) (data not shown). To verify the phenotype of the ATMT mutant, *bcsep1* was deleted by a replacement approach (for details, see Materials and Methods). In sum, two independent deletion mutants were obtained that lack *bcsep1* and additional integrations of the replacement cassette (data not shown). As the ATMT mutant, Δ*bcsep1* mycelia were unable to colonize bean leaves (Fig. 10B); though, necrotic spots beneath the mycelial plugs occurred that were likely due to the hypersensitive response of the plant (data not shown). However, Δ*bcsep1* mutants were already severely impaired in growth on solid media forming small compact colonies and were not able to differentiate any reproductive structures (conidia and sclerotia) (Fig. 10C). Microscopic analyses of the slowly growing Δ*bcsep1* hyphae revealed increased apical branching rates and CFW (calcofluor white) staining demonstrated the absence of septa (Fig. 10D). Taken together, the formin BcSep1 is essential for establishment of normal polarized growth (with a dominant hyphal axis) and septum formation and consequently for subsequent differentiation processes including the formation of a hyphal network and reproductive structures.

### BcFar1 has no obvious functions

In *S. cerevisiae*, the C3HC4-type ring zinc finger domain-containing Far1p specifies the direction of polarized growth during mating by linking the Gβγ dimer to the polarity establishment proteins Cdc24p, Bem1p, and Cdc42p [33]. BlastP analyses in the *B. cinerea* Database using the sequence of Far1p as query revealed a single hit (hereafter referred as *bcfar1*); however, overall sequence similarity is very low as only the N-terminal C3HC4-type RING domain is conserved in both protein sequences. Apart from this RING domain (IPR001841) spanning aa 134–186, BcFar1 contains a pleckstrin homology (PH; IPR001849) (aa 428–566) and a von Willebrand factor type A (VWA; IPR002035) domain (aa 632–812) as its orthologs in filamentous fungi such as *A. nidulans* AN5167 and *N. crassa* NCU07565 which exhibit 59% and 68% aa identity with BcFar1. To our knowledge, the role of Far1-like proteins of filamentous fungi has not been addressed experimentally so far. Therefore, we generated deletion mutants to study whether BcFar1 has impact on hyphal growth and associated processes (for details, see Material and Methods). However, we failed to identify any significant phenotypes (data not shown) suggesting that the Far1-like protein in *B. cinerea* is not involved in establishment of polarized growth as its counterpart in budding yeast.

## Discussion

The role of the scaffold protein Bem1p as a major structural component of the polarity complex has been studied in detail in yeast, whereas only limited information is currently available for filamentous fungi. Compared to a recent analysis in the saprophytic model systems *A. nidulans* (BemA) and *N. crassa* (Bem-1) [15], [17], our detailed functional characterization of the Bem1 ortholog in the necrotrophic pathogen *B. cinerea* revealed parallels, but also significant differences. In *N. crassa* and *B. cinerea*, Bem1 does not seem to have significant impact on hyphal morphology and polar growth while Δ*bemA* hyphal tips are swollen. In contrast to *N. crassa*, BemA and BcBem1 are not even necessary for normal radial growth rates. Other parallels of *bem1* deletion mutants include significantly reduced conidiation rates and the formation of malformed conidia with increased numbers of nuclei by Δ*bem-1* and Δ*bcbem1* mutants, suggesting an impact of Bem1 orthologs on the cell cycle. However, as in *N. crassa* but unlike *A. nidulans*, in *B. cinerea* the conidial germination is impaired in the Δ*bcbem1* mutant. Both BcBem1 and Bem-1 are involved in the formation of CATs; however, in contrast to the situation in *N. crassa*, BcBem1 seems essential for the process as the deletion mutant does not form CAT fusions at all. In contrast to the saprophytes *N. crassa* and *A. nidulans*, in the endophyte *E. festucae* a *bem1* deletion mutant showed significant disturbance in vegetative differentiation (hyphal morphogenesis, CAT formation) and growth in culture and *in planta* [16], [18]. Here to our knowledge for the first time the impact of a Bem1 ortholog on the differentiation steps and developmental switches associated with virulence in a phytopathogenic fungus is described: in *B. cinerea* BcBem1 affects differentiation of appressoria and penetration hyphae, and growth *in planta*. As already indicated by the hydrophobic surface germination test, BcBem1 seems to be involved in surface sensing and - probably linked - in development of functional penetration structures. *B. cinerea* forms two morphologically different penetration structures. The first are appressoria, terminal singular hyphal swellings of conidial germination tubes, which firmly attach to the surface. Since these structures seem to lack the normal sealing septum in *B. cinerea* they were called appressoria-like structures [34]. However, recently we could show that the tetraspanin BcPls1 (required for functional appressoria) is exclusively formed in the appressoria and the adjacent short hyphal fragment till the first regular septum, strongly suggesting that this “normal” septum takes over the sealing function, which would allow e.g. also the generation of pressure [35]. The second type of penetration structures are infection cushions, which are highly branched hyphae originating from already established mycelial networks [36]. The two processes seem to be differently regulated: analyses of the NADPH oxidase (Nox) complexes in our lab indicated that the catalytic subunit BcNoxA is necessary for formation of infection cushions on glass, whereas BcNoxB is required for formation of functional appressoria ([35] and unpubl. data). The Δ*bcbem1* mutant shows an intermediate phenotype (regular branching before appressoria formation), indicating that the normal germ tube-associated program of appressoria differentiation is disturbed. This (and the observed slower lesion development in early stages) links BcBem1 to the BcNoxB complex, whereas the strong impact on CAT formation is indicative for a link to BcNoxA (see below). The slower colonization of host tissue by the Δ*bcbem1* mutant could be just due to the delay of germination; however, the infection test with mycelial plugs shows a comparable delay in colonization of plant tissue as seen for infection with conidia.

Taken together, our data show that BcBem1 is involved in all major differentiation processes in *B. cinerea* requiring polar growth/redirection of growth, but not in normal hyphal growth. As discussed in detail in Schürg et al. [17], this could indicate that hyphal growth is mainly dependent on a direct link between the terminal complexes and the cytoskeleton, i.e. positive cytoskeleton-based feedback loops, whereas special differentiation steps require soluble signaling-based loops including Bem1 [37]. The observed strong effect of the formin BcSep1 (which forms a link to cytoskeleton organization) on growth supports this hypothesis. Also the localization studies using reporter gene fusions substantiate this idea: in contrast to yeast and e.g. *N. crassa* (where Bem1 is required for normal growth rate [17]) we rarely observed a tip localization of BcBem1 in “normal” hyphae. The regular association of BcBem1 to septa (which was also observed in *N.crassa* [17]) suggests a function in the formation of septa. This process is not yet fully understood in hyphal fungi [38]; among others landmark proteins, actin-binding proteins, a GTPase complex, and septins cooperate to form an actomyosin ring. The interaction between BcBem1 and the *B. cinerea* formin BcSep1 (see below) supports this possible role of BcBem1 in septum formation. However, as in *N. crassa* BcBem1 is not essential for this process, in contrast to BcSep1. It is possible that BcBem1 supports (but is not absolutely required for) the localization of BcSep1 at the septum-building site. Localization approaches *in planta* or on onion epidermis were not yet successful because of the strong background fluorescence; during CAT formation no tip localization was detected.

The role in so many different developmental processes indicates that BcBem1 is highly flexible and can cooperate with different partners. Phenotypic overlaps to various signaling mutants described in *B. cinerea* indicate links to a wide range of complex partners/signaling cascades. Very similar is also the Δ*bccdc42* mutant [14]: it is impaired in conidiation, forms very large conidia with many nuclei, which show retarded germination, and also the effect on virulence is comparable. This fits to the observed strong interaction of BcBem1 with BcCdc24, which has been shown also in filamentous fungi to be a GEF for Cdc42 [23]. However, there are also significant differences: Δ*bccdc42* does not form any sclerotia; in contrast, it does sporulate also in darkness, i.e. it seems to be blind; and it can form CATs, though less effective than the WT (U. Siegmund, unpubl. data). Obviously, BcBem1 interacts in some developmental steps with the Cdc42-polarity complex, but in others there must be different interaction partners. There is also some overlap with phenotypes of mutants impaired in the BcNox complexes, but not a clear correlation with one of the two complexes (BcNoxA/B) (see above); in addition, both complexes have, in contrast to BcBem1, positive (BcNoxA) or neutral (BcNoxB) input on sclerotia formation [35], [39]. It has been shown in the endophyte *E. festucae* that Bem1 interacts with the regulatory subunit of the Nox complexes [16]; there is good evidence that Bem1 actually might be the fungal equivalent of the phagocytic scaffold protein p40^phox^ which plays a major role in Nox complex assembly [13], [16]. Whereas an interaction of the Bem1 ortholog with the regulatory subunit NoxR in *C. purpurea*, a close relative of *E. festucae*, could be confirmed[40], we could not show such an interaction in *B. cinerea*. Therefore, the exact role of BcBem1 on recruitment and assembly of the Nox complexes in *B. cinerea* remains to be resolved.

Schürg et al. [17] described an effect of Bem1 on activation of the MAP kinase Mak2, an ortholog of the yeast Fus3p MAPK, which is involved in CAT formation. In *B. cinerea* a connection between the corresponding Fus3 ortholog, Bmp1, and BcBem1 is not obvious. Bmp1 mutants are also impaired in penetration, but in contrast to BcBem1 the process of appressoria formation is totally blocked; and the germination of conidia is only affected on a hydrophobic surface [20], [41]. More phenotypic overlap with Δ*bcbem1* is evident for mutants lacking Bmp3, a homolog of the yeast MAPK Slt2p involved in the cell integrity pathway [42]: Δbmp3 mutants show prolonged germ tubes and retarded penetration. Slt2p is also related to the regulation of actin cytoskeleton polarization in yeast [43] and to the mitotic delay induced by actin cytoskeleton perturbation as part of the morphogenesis checkpoint mechanism [44], thus a link to the polarity complex and BcBem1 in *B. cinerea* is likely and deserves further investigations.

According to the described function of Far1p in yeast in recruiting the Gβγ dimer of heterotrimeric G proteins to the polarity complex (Bem1p-Cdc24p-Cdc42p), we investigated the function of the *B. cinerea* ortholog BcFar1. Due to the lack of obvious Δ*bcfar1* phenotypes, we suggest that the protein is not involved in BcBem1-related processes. However, the Gβγ dimer in *B. cinerea* affects cell polarity as well: in loss-of-function mutants the axis of hyphal growth is totally straight, distances between septa are larger and fewer subapical branching events occur (unpublished data).

As a direct interaction partner of BcBem1 we could so far identify unequivocally only BcCdc24 and BcSep1. The strong association of Bem1 and Cdc24 has also been shown e.g. in *U. maydis*, where Bem1 obviously is involved in recruitment of Cdc24 to the hyphal tip [45]. As in most other fungi [16], [22], [45], [46], the Δ*bccdc24* mutation seems to be lethal as no homokaryotic strains could be obtained. Cdc24 plays a central role in establishment and maintenance of polarity; its strong effect on hyphal growth is probably due to the fact that it can serve as a GEF for Cdc42 and for Rac, as has been demonstrated by *in vitro* activation assays for *N. crassa* [23] and *C. purpurea* [22]. In *B. cinerea*, both GTPases have been characterized. Obviously, BcRac has more essential functions than BcCdc42 as Δ*bcrac* mutants are severely impaired in hyphal morphology and fail to differentiate conidia and sclerotia and to infect [47].

As another interaction partner of BcBem1 we identified the single formin in *B. cinerea* (BcSep1) that is the ortholog of yeast Bni1p. As described for *N. crassa* [31], *B. cinerea* lacks an ortholog of the second formin found in yeast (Bnr1p, Bni1p-related 1). Deletion mutants are viable, but severely affected in hyphal growth and all associated processes. In common with *A. nidulans* SepA [29], [48] and *N. crassa* Bni-1 [31], BcSep1 is essential for septum formation and has strong impact on polarized growth indicating that functions of this formin are conserved in filamentous fungi. In agreement with their functions, SepA and BNI-1 localize to the sites of polarized growth, to sites of septation, the hyphal tips and the sites of cell fusions [30], [31]. Formins are well-known effector proteins of Rho GTPases participating in actin cytoskeleton remodeling through regulation of actin filament assembly [25]. Hyphal and colony morphology of Δ*bcsep1* and Δ*bcrac* mutants are similar; however, Δ*bcrac* hyphae contain septa. Nevertheless, the impact of BcRac on the subcellular actin distribution is evident [47]. Similarly to Δ*bcsep1* mutants, the inhibition and deletion of the calcineurin phosphatase (BcCnA) result in small compact undifferentiated colonies formed by non- or irregularly septated and hyper-branched hyphae [49] suggesting furthermore the involvement of Ca^2+^/calcineurin-dependent signaling in establishment and maintenance of polarity of hyphae of *B. cinerea*.

## Materials and Methods

### *B. cinerea* strains and growth conditions

Strain B05.10 of *B*. *cinerea* Persoon:Fries is an isolate from *Vitis vinifera* (Table 1) and is used as recipient strain for genetic modifications. The genome sequence of B05.10 was published [50] and recently updated by Staats and van Kan [51]. *B. cinerea* strains were cultivated on plates containing solid synthetic complete medium (CM) [52], Gamborg B5 ( = GB5) +2% glucose (Duchefa, The Netherlands), modified Czapek-Dox (CD) as minimal medium (2% sucrose, 0.1% KH_2_PO_4_, 0.3% NaNO_3_, 0.05% KCl, 0.05% MgSO_4_ x 7 H_2_O, 0.002% FeSO_4_ x 7 H_2_O, pH 5.0), or solid potato dextrose (PDA) agar (Sigma-Aldrich, Germany) supplemented with 10% homogenized bean leaves (PDAB). Cultures were incubated at 20°C under white light (12 h light/12 h darkness) for conidiation, and in continuous darkness for induction of sclerotia formation. For DNA isolation, mycelia were grown on solid CM with cellophane overlays.

### Standard molecular methods

Fungal genomic DNA was prepared according to Cenis [53]. For Southern blot analysis, genomic DNA was digested with restriction enzymes (Fermentas, Germany), separated on 1% (w/v) agarose gels and transferred to Amersham Hybond-N+ filters (GE Healthcare Limited, UK) according to Sambrook et al. [54]. Blot hybridizations with random-primed α-^32^P-dCTP-labelled probes were performed as described previously [55]. PCR reactions were performed using the high-fidelity DNA polymerase Phusion (Finnzymes, Finland) for cloning purposes and the BioTherm Taq DNA Polymerase (GeneCraft, Germany) for diagnostic applications. Replacement fragments and expression vectors were assembled in *Saccharomyces cerevisiae* by exploiting its homologous recombination machinery [56], [57]. Sequencing of DNA fragments was performed with the Big Dye Terminator v3.1 sequencing kit (Applied Biosystems, USA) in an ABI Prism capillary sequencer (model 3730; Applied Biosystems). For sequence analysis, the program package DNAStar (Madison, USA) was used. Protocols for protoplast formation and transformation of *B. cinerea* were described by Schumacher [57]. Regenerated protoplasts were overlaid with SH agar containing 70 µg/ml hygromycin B (Invitrogen, The Netherlands) and 100 µg/ml nourseothricin (Werner-Bioagents, Germany), respectively. Resistant colonies were transferred to agar plates containing CM medium supplemented with the respective selection agent (70 µg/ml). To obtain homokaryotic strains, conidial suspensions were spread on selective media, or protoplasts were embedded in selective SH agar.

### Generation of *B. cinerea* mutants

For replacement of *bcbem1*, its 5′ and 3′-non-coding regions were linked with a hygromycin cassette (P*oliC*::*hph*) by conventional cloning in *E. coli*. Flanking sequences (1,000 bp and 1,092 bp) were amplified using primer pairs *bcbem1*-LFF/LFR and *bcbem1*-RFF/RFR, digested with KpnI/SalI and EcoRI/XhoI (Table S1, Fig. S1B), and inserted into the corresponding restriction sites of pOliHP [58] creating pΔ*bcbem1*. For transformation, the replacement fragment was isolated by digestion with KpnI and XhoI. Homologous integration events in hygromycin-resistant transformants were detected by diagnostic PCR using the primers pLOF-oliP and pAN-T, binding in the resistance cassette and the primers *bcbem1*-LFF2 and *bcbem1*-Rdia binding upstream and downstream of the *bcbem1*-flanking regions, respectively. Single spore isolates were screened for the absence of *bcbem1* alleles using primers *bcbem1*-LFF2 and *bcbem1*-RIntr (Fig. S1C). For Southern blot analysis, genomic DNA was digested with XbaI, blotted, and hybridized with the 5′ flank of the replacement fragment (Fig. S1D). The complementation vector was cloned by yeast recombinational cloning. For that, *bcbem1* including 1,008 bp of the promoter region was amplified using primers *bcbem1*-Com-F and Com-R and co-transformed into *S. cerevisiae* FY834 with the SpeI/NotI-digested plasmid pNAN-OGG [57] resulting in p*bcbem1*-COM. Similarly, the vector for expression of a GFP-BcBem1 fusion protein was generated. The ORF of *bcbem1* was amplified using primers *bcbem1*-GFP-F and *bcbem1*-GFP-R and assembled in yeast with the NotI-digested pNAN-OGG yielding pNAN-PoliC::GFP-BcBem1. Generated constructs for complementation and GFP fusion comprise gene flanks of *bcniiA* (nitrite reductase) for targeted integration at the respective gene locus and a nourseothricin resistance cassette (P*trpC*::*nat1*) and were transformed into Δ*bcbem1* (T18). Homologous integration of the constructs at *bcniiA* was verified by diagnostic PCR using primer pairs *bcniiA*-hi5F/T*gluc*-hiF (*bcniiA*-5′) and *bcniiA*-hi3R/*nat1*-hiF (*bcniiA*-3′), respectively. Replacement fragments for *bccdc24*, *bcsep1* and *bcfar1* were generated by yeast recombinational cloning following the same scheme: amplifications of 5′- and 3′-non-coding regions using primer pairs 5F/5R and 3F/3R, amplification of the P*trpC*::*hph* cassette from pCSN44 using primers *hph-*F and *hph*-R (Table S1), and assembly of fragments in *S. cerevisiae* FY834. For transformation of *B. cinerea* B05.10, replacement fragments were amplified using primer pairs 5F/3R and the isolated plasmids (pΔ*bccdc24*, pΔ*bcsep1*, pΔ*bcfar1*) as template. Homologous integration events in hygromycin-resistant transformants were detected by diagnostic PCR using primer pairs hi5F/TrpC-T (at 5′) and hi3R/TrpC-P2 (at 3′). In total, two transformants that have undergone homologous integration at the respective gene locus were obtained for *bccdc24*, *bcsep1* and *bcfar1*. For these, Southern blot analyses were employed to verify the absence of additional, ectopic integrations of the replacement constructs (data not shown). Homokaryotic strains for Δ*bcsep1* (T4, T8) and Δ*bcfar1* (T1.5, T1.6) were generated by single conidium/protoplast isolation. Generation of homokaryotic Δ*bccdc24* mutants failed.

### Germination and germling fusion assays

For analyses of nutrient-dependent germination on glass surfaces [20], conidia were collected, washed and resuspended in water. 25-µl droplets of conidial suspensions (5×10^5^/ml) were placed on cover slips in 24-well plates and flooded with 475 µl of GB5 solution supplemented with glucose, fructose or xylose (10.5 mM). Incubation took place in darkness at 20°C. Germination progress was monitored by light microscopy after 3, 6 and 24 h. For hydrophobicity-induced germination (in absence of nutrients), conidia were prepared as described above. 25-µl droplets (10^5^/ml) were placed onto polypropylene foil. Germination was monitored after incubation for 24 h in darkness at 20°C.

The ability to form conidial anastomosis tubes (CATs) and vegetative hyphal fusions was tested as described by Roca and Weichert *et al*. [21]. 300 µl of conidial suspensions (1×10^7^/ml H_2_O) were dispersed on solid Vogel's minimal medium [59] and incubated for 14-16 h. Samples were analyzed using differential interference contrast (DIC) microscopy. For quantification a minimum of three independent replicates were performed evaluating at least 300 conidia each time.

### Infection assays

Pathogenicity assays were performed with conidia harvested from 7- to 10-d-old agar cultures. Primary leaves of *Phaseolus vulgaris* L. genotype N90598 (originating from J. D. Kelly, Michigan State University, East Lansing, MI) were inoculated with 7.5 µl of conidial suspensions (2×10^5^/ml in GB5 +2% glucose) as described by Klimpel *et al*. [60]. Infected plants were incubated in humid chambers at 20°C under natural illumination conditions. For penetration assays, onion epidermal strips were peeled, washed with water and incubated at 70°C for 1 h in a humid chamber. Strips were inoculated with 10-µl droplets of conidial suspensions (5×10^4^/ml in water) and incubated for 24 h under humid conditions. Before light microscopy, samples were treated with lactophenol aniline blue (Sigma-Aldrich, Germany) to stain fungal structures that are located on the surface of the epidermis.

### Light and epifluorescence microscopy

For microscopy, 10-µl droplets of conidial suspensions (1×10^5^/ml in GB5 +2% glucose) were placed on glass slides and incubated at room temperature in humid conditions. Microscopy was performed 12–24 hpi with AxioImager.M1 using 63x or 40x objective lens or ObserverZ.1 using a 20x objective lens (Zeiss, Jena, Germany). When indicated, differential interference microscopy (DIC) was used for bright field images. Nuclei were stained with Hoechst 33342 (Frankfurt, Germany) according to Kangatharalingam and Ferguson [61]; and cell wall material with calcofluor white (CFW) (Sigma-Aldrich, Germany). Specimens stained by Hoechst 33342 and CFW were examined using the filter set 49 DAPI shift free (excitation G 365, beam splitter FT 395, emission BP 445/50). GFP fluorescence was detected with filter set 38 (excitation BP 470/40, beam splitter FT 495, emission BP 525/50). Images were captured with a Zeiss AxioCamMRm camera and analyzed using the AxiovisionRel 4.8 software package.

### Yeast two-hybrid assays

Protein-protein interaction assays were performed according to the standard yeast two-hybrid method established by Fields and Song [62]. Proteins of interest were fused to Gal4 activation and DNA binding domains in vectors pAD-GAL4-2.1 and pBD-GAL4 Cam (Stratagene Corp., CA, USA), respectively. Vector cloning was accomplished in yeast using strain SMY3 as host (-leu-trp) [63]. For that, genes of interest were amplified with primers containing overlaps with the Gal4 activation (AD-F, AD-R) or binding domain (BD-F, BD-R) using cDNA as template. Amplicons were co-transformed with SalI-digested plasmids pAD-GAL4-2.1 (selection on SD-leu) and pBD-GAL4-Cam (selection on SD-trp), respectively. Assembled vectors were isolated and transformed into *E. coli* for amplification and sequencing. For interaction assays, *S. cerevisiae* strain PJ69-4A [64] was used and transformed according to a modified protocol of the lithium acetate method [65]. Selection took place on SD -leu-trp for obtaining strains with both vectors. For drop tests, the strains were grown over night in 5 ml of liquid SD and adjusted to an OD (600 nm) of 1. Cells were starved in 1 M sorbitol for 5 h at 30°C and serial dilutions of 1∶10, 1∶100, and 1∶1000 were prepared. Dilutions (10 µl) were spotted on solid SD-leu-trp and SD-leu-trp-his+X-Gal medium (if needed supplemented with 3-AT (3-Amino-1,2,4-triazole)). General growth of the strains after 3–4 d at 30°C was evident on SD-leu-trp medium, whereas interaction of prey and bait proteins was indicated by growth and blue staining on SD-leu-trp-his+X-Gal medium. pAD-CpRac^G17V^/pBD-CpCla4 (*C. purpurea*) were used as a positive control [66].

### Database resources

Nucleotide and protein sequences of *B. cinerea* strain B05.10 were extracted from the *B. cinerea* Database (Broad Institute; http://www.broadinstitute.org/annotation/genome/botrytis_cinerea/). BLAST analyses were performed using the databases of the National Center for Biotechnology Information (NCBI)

(http://blast.ncbi.nlm.nih.gov/Blast.cgi). Conserved protein domains were predicted by InterProScan (http://www.ebi.ac.uk/interpro/).

## Supporting Information

Figure S1 Construction of *bcbem* deletion mutants. (**A**) Domain structure of BcBem1. SH3 - Src homology 3 domain (IPR001452), PX - phox homologous domain (IPR001683), PB1 - Phox/Bem1p domain (IPR000270). (**B**) *Bcbem1* was replaced by a hygromycin resistance cassette. Primers used for diagnostic PCR are indicated by arrows. (**C**) Diagnostic PCR revealed the absence of *bcbem1* (exemplarily shown for one homokaryotic mutant). (**D**) Southern blot analysis demonstrated the absence of additional integration events of the replacement construct. Genomic DNA was digested with XbaI, transferred to a nylon membrane and hybridized with the 5′ flank (dotted line in B). A smaller hybridizing fragment in Δ*bcbem1* is due to the additional XbaI restriction site in the hygromycin resistance cassette. For more details see Materials and Methods.
(TIF)

Figure S2 Virulence defect of Δ*bcbem1* mutants is not restricted to the use of conidia as inoculum. Primary leaves of *P. vulgaris* were inoculated with plugs of non-sporulating mycelia of wild type and Δ*bcbem1*. Mean values and standard deviations were calculated from 12 lesions per strain with two measurements per lesion.
(TIF)

Table S1 Oligonucleotides used in this study.
(DOCX)
