# Supplementary figures and images for: Functional Analysis of BcBem1 and Its Interaction Partners in Botrytis cinerea: Impact on Differentiation and Virulence
Source: PLoS One. 2014 May 5;9(5):e95172. doi: 10.1371/journal.pone.0095172 (PMC4010548; doi:10.1371/journal.pone.0095172)

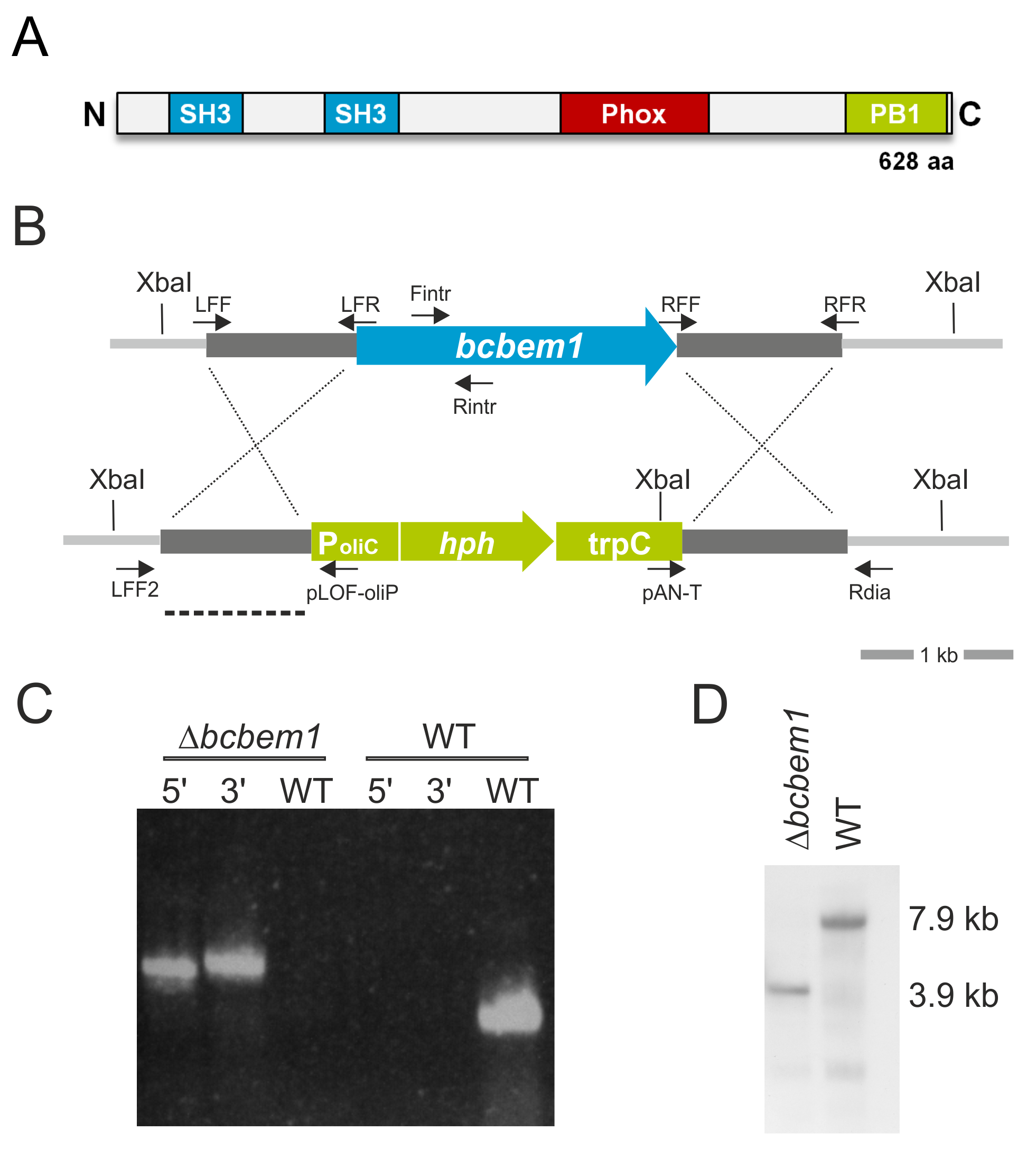

Supplement: Figure S1 — Construction of bcbem deletion mutants. (A) Domain structure of BcBem1. SH3 - Src homology 3 domain (IPR001452), PX - phox homologous domain (IPR001683), PB1 - Phox/Bem1p domain (IPR000270). (B) Bcbem1 was replaced by a hygromycin resistance cassette. Primers used for diagnostic PCR are indicated by arrows. (C) Diagnostic PCR revealed the absence of bcbem1 (exemplarily shown for one homokaryotic mutant). (D) Southern blot analysis demonstrated the absence of additional integration events of the replacement construct. Genomic DNA was digested with XbaI, transferred to a nylon membrane and hybridized with the 5′ flank (dotted line in B). A smaller hybridizing fragment in Δbcbem1 is due to the additional XbaI restriction site in the hygromycin resistance cassette. For more details see Materials and Methods. (TIF) [file pone.0095172.s001.tif]

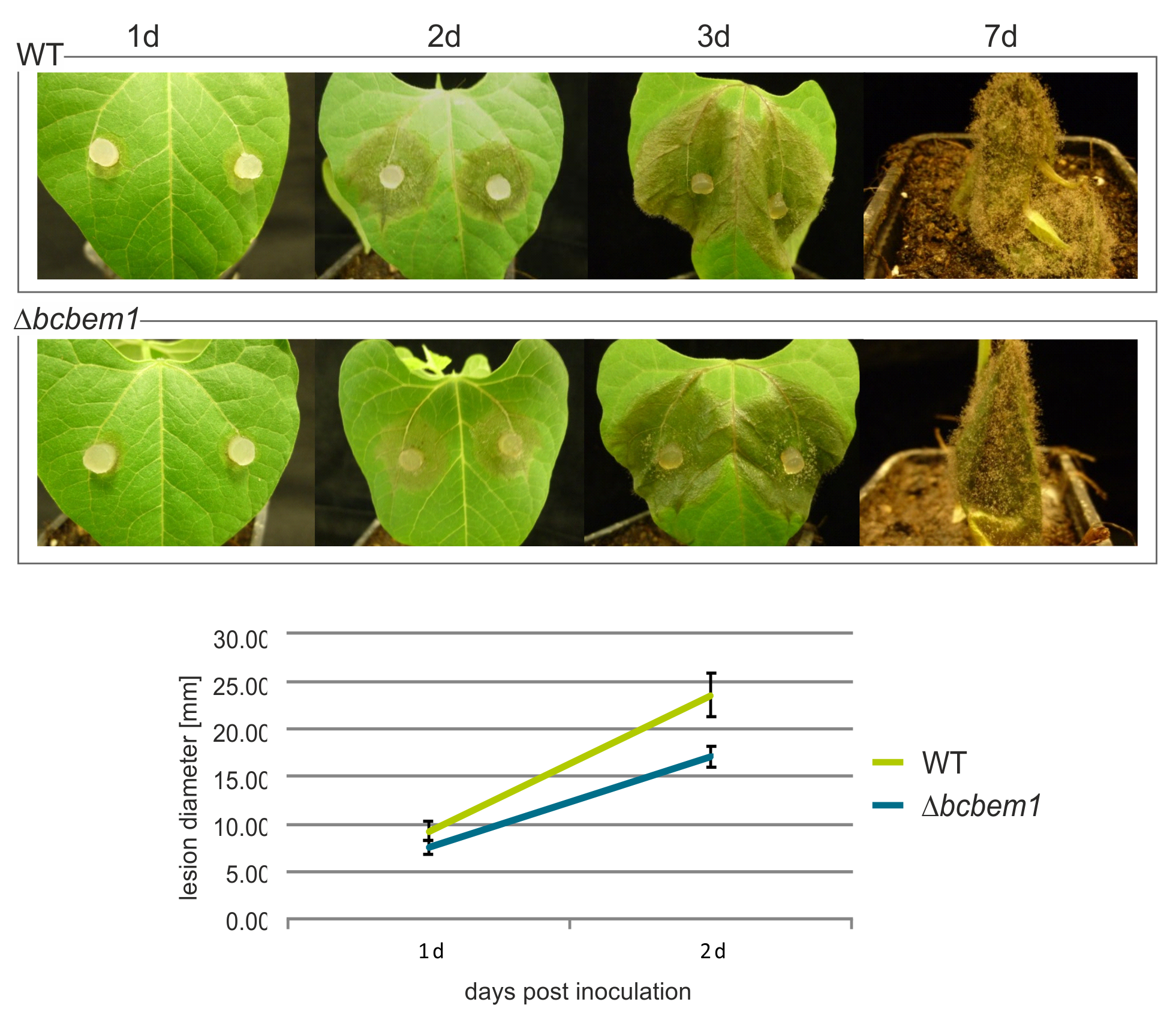

Supplement: Figure S2 — Virulence defect of Δbcbem1 mutants is not restricted to the use of conidia as inoculum. Primary leaves of P. vulgaris were inoculated with plugs of non-sporulating mycelia of wild type and Δbcbem1. Mean values and standard deviations were calculated from 12 lesions per strain with two measurements per lesion. (TIF) [file pone.0095172.s002.tif]
