# Supplementary material for: Functional Analysis of BcBem1 and Its Interaction Partners in Botrytis cinerea: Impact on Differentiation and Virulence
Source: PLoS One. 2014 May 5;9(5):e95172. doi: 10.1371/journal.pone.0095172 (PMC4010548; doi:10.1371/journal.pone.0095172)
Supplement: Table S1 — Oligonucleotides used in this study. (DOCX) [file pone.0095172.s003.docx]

**Table S1. Oligonucleotides used in this study.**

| **Primer** | **Sequence (5’ ⭢ 3’)** | **Features** | **Used for** |
| --- | --- | --- | --- |
| ***Bcbem1*-LFF** | GGTACCGGCCGGAGAACAGTCCCTTTTC | *Bcbem1*-5’ | Cloning – amplification of 5' flank for Δ*bcbem1* construct |
| ***Bcbem1*-LFR** | GTCGACGAGAGTGGATGTAAAT | *Bcbem1*-5’ | Cloning – amplification of 5' flank for Δ*bcbem1* construct |
| ***Bcbem1*-RFF** | GAATTCCATGTATCTGGTACTGCACTGGC | *Bcbem1*-3’ | Cloning – amplification of 3' flank for Δ*bcbem1* construct |
| ***Bcbem1*-RFR** | CTCGAGCTCGCCCTGGAACAAAC | *Bcbem1*-3’ | Cloning – amplification of 3' flank for Δ*bcbem1* construct |
| ***Bcbem1*-Rdia** | GCCTCCGTATGCGCAGTGGAGG | *Bcbem1*-3’ | Diagnostic PCR ∆*bcbem1* – homologous integration at 5' |
| ***Bcbem1*-LFF2** | GGTACCGGAAGTAGTCCCTTGCCCC | *Bcbem1*-5’ | Diagnostic PCR ∆*bcbem1* – homologous integration at 3' |
| **pLOF-oliP** | GGTACTGCCCCACTTAGTGGCAGCTCGCG | P*oliC* | Diagnostic PCR Δ*bcbem1* – homologous integration at 5' |
| **pAN-T** | CCCAGAATGCACAGGTACAC | T*trpC* | Diagnostic PCR Δ*bcbem1* – homologous integration at 3' |
| ***Bcbem1*-RIntr** | CCCCTTCGCGAGGTGCAAAG | *Bcbem1* ORF | Diagnostic PCR ∆*bcbem1* – detection of *bcbem1* alleles |
| ***Bcbem1-*Com-F** | gcccaaaaaatgctccttcaatatc-GCCATATTCAACAGTAGAAACGGCTC | P*trpC – bcbem1*-5’ | Cloning – amplification of *bcbem1* locus for complementation construct |
| ***Bcbem1-*Com-R** | gtctgcatacgaaaataccccacg-GGTGGTGATCTGCGTCAAAATGAG | *BcniiA*-5’ *– bcbem1*-3’ | Cloning – amplification of *bcbem1* locus for complementation construct |
| ***Bcbem1-*GFP-F** | gggaatggatgaactttacaaa-ATGTTTGTCTATCAGGCTCTTC | *Gfp* *– bcbem1* | Cloning – amplification of *bcbem1* locus for GFP fusion construct |
| ***Bcbem1-*GFP-R** | catacatcttatctacatacg-CTATTGATATTCGACGTAAATAATAAG | T*gluc* *– bcbem1* | Cloning – amplification of *bcbem1* locus for GFP fusion construct |
| ***Bcbem1*-Y2H-F1** | gaattcATGTTTGTCTATCAGGCTCTTCG | EcoRI *– bcbem1* | Cloning – amplification of *bcbem1* for integration in both Y2H vectors |
| ***Bcbem1*-Y2H-R1** | gtcgacTTGATATTCGACGTAAATAATAAGCTTATCGTTCCG | SalI *– bcbem1* | Cloning – amplification of *bcbem1* for integration in both Y2H vectors |
| ***Hph*-F** | GTCGGAGACAGAAGATGATATTGAAGGAGC | *PtrpC* | Cloning – amplification of P*trpC*::*hph* for Δ*bcsep1*, Δ*bccdc24,* Δ*bcfar1* |
| ***Hph*-R** | GTTGGAGATTTCAGTAACGTTAAGTGGAT | *Hph* | Cloning – amplification of P*trpC*::*hph* for Δ*bcsep1*, Δ*bccdc24,* Δ*bcfar1* |
| ***TrpC*-P2** | CCTCCACTAGCTCCAGCCAAGCCC | P*trpC* | Diagnostic PCR Δ*bcsep1*, Δ*bccdc24* – homologous integration at 5' |
| ***TrpC*-T** | GGAATAGAGTAGATGCCGACCGG | T*trpC* | Diagnostic PCR Δ*bcsep1*, Δ*bccdc24* – homologous integration at 3' |
| ***Bcsep1*-5F** | gtaacgccagggttttcccagtcacgacg-CCGGGCCAGCCCACCACCCC | pRS426-5F – *bcsep1*-5’ | Cloning – amplification of 5' flank for Δ*bcsep1* construct |
| ***Bcsep1*-5R** | atccacttaacgttactgaaatctccaac-CTTCGTGTCCCAATACTTCCC | *Hph – bcsep1*-5’ | Cloning – amplification of 5' flank for Δ*bcsep1* construct |
| ***Bcsep1*-3F** | ctccttcaatatcatcttctgtctccgac-GAGGGACTGTAGATTATGGGG | *PtrpC – bcsep1*-3’ | Cloning – amplification of 3' flank for Δ*bcsep1* construct |
| ***Bcsep1*-3R** | gcggataacaatttcacacaggaaacagc-CCCCCGCTCACACTCGAGCC | pRS426-3R – *bcsep1*-3’ | Cloning – amplification of 3' flank for Δ*bcsep1* construct |
| ***Bcsep1*-hi5F** | CGGCCGCCACAGGTTCCTTATC | *Bcsep1*-5’ | Diagnostic PCR Δ*bcsep1* – homologous integration at 5' |
| ***Bcsep1*-hi3R** | CATCCACCCAATCACTCCTCCCC | *Bcsep1*-3’ | Diagnostic PCR Δ*bcsep1* – homologous integration at 3' |
| ***Bcsep1*-WT-F** | CGCGGCGAGTGGTAGAGGATC | *Bcsep1* ORF | Diagnostic PCR Δ*bcsep1* – detection of *bcsep1* alleles |
| ***Bcsep1*-WT-R** | CCATTCCGGGGTTCCTTCTTCC | *Bcsep1* ORF | Diagnostic PCR Δ*bcsep1* – detection of *bcsep1* alleles |
| ***Bcsep1*-AD-F** | gaattaggatcctctgctagcaga*gaattc*-ATGTCGTCAACTGGCAAATCCTC | pAD-EcoRI *– bcsep1* | Cloning – amplification of *bcsep1* for integration in pAD-GAL4-2.1 |
| ***Bcsep1*-AD-R** | cagtatctacgattcatagatctc*gtcgac*-TCACTCATCCAACTCAATCGGTTT | pAD-SalI *– bcsep1* | Cloning – amplification of *bcsep1* for integration in pAD-GAL4-2.1 |
| ***Bcsep1*-AD-RK** | cagtatctacgattcatagatctc*gtcgac*-CCAAGCATCGAACCGTCCAT | pAD-SalI *– bcsep1* | Cloning – amplification of *bcsep1* for integration in pAD-GAL4-2.1 |
| ***Bcsep1*-BD-F** | caaagacagttgactgtatcgccg*gaattc*-ATGTCGTCAACTGGCAAATCCTC | pBD-EcoRI *– bcsep1* | Cloning – amplification of *bcsep1* for integration in pBD-GAL4 Cam |
| ***Bcsep1*-BD-R** | ttcgcccggaattagcttggctgc*gtcgac*-TCACTCATCCAACTCAATCGGTTT | pBD-SalI *– bcsep1* | Cloning – amplification of *bcsep1* for integration in pBD-GAL4 Cam |
| ***Bcsep1*-BD-RK** | ttcgcccggaattagcttggctgc*gtcgac*-CCAAGCATCGAACCGTCCAT | pBD-SalI *– bcsep1* | Cloning – amplification of *bcsep1* for integration in pBD-GAL4 Cam |
| ***Bccdc24*-5F** | gtaacgccagggttttcccagtcacgacg-GGGAGCGAAAGGAACGGAGGAG | pRS426-5F – *bccdc24*-5’ | Cloning – amplification of 5' flank for Δ*bccdc24* construct |
| ***Bccdc24*-5R** | atccacttaacgttactgaaatctccaac-CCGGGTTGAACTGCAGGTGGC | *Hph – bccdc24*-5’ | Cloning – amplification of 5' flank for Δ*bccdc24* construct |
| ***Bccdc24*-3F** | ctccttcaatatcatcttctgtctccgac-CTGGAAGGGGGATCCAGGTG | pRS426-3R – *bccdc24*-3’ | Cloning – amplification of 3' flank for Δ*bccdc24* construct |
| ***Bccdc24*-3R** | gcggataacaatttcacacaggaaacagc-CTGGGATCTGGGAACGCCAGC | *PtrpC – bccdc24*-3’ | Cloning – amplification of 3' flank for Δ*bccdc24* construct |
| ***Bccdc24*-HI-F1** | CCACGTACTTGGCCCAGATAGGC | *Bccdc24-*5’ | Diagnostic PCR Δ*bccdc24* – homologous integration at 5' |
| ***Bccdc24*-HI-R1** | GCGGTGGCTGCTGCTGTTGCAG | *Bccdc24-*3’ | Diagnostic PCR Δ*bccdc24* – homologous integration at 3' |
| ***Bccdc24*-WT-R** | GCTGGCTGGAGGGTATTGTAGAGC | *Bccdc24* ORF | Diagnostic PCR Δ*bccdc24* – detection of *bccdc24* alleles |
| ***Bccdc24-*AD-F** | gaattaggatcctctgctagcaga*gaattc*-ATGTCATCTGGCGGTGTTGCCATT | pAD-EcoRI *– bccdc24* | Cloning – amplification of *bccdc24* for integration in pAD-GAL4-2.1 |
| ***Bccdc24-*AD-R** | cagtatctacgattcatagatctc*gtcgac*-TTAACGCTCGCCGTCAATGCTTAA | pAD-SalI *– bccdc24* | Cloning – amplification of *bccdc24* for integration in pAD-GAL4-2.1 |
| ***Bccdc24-*BD-F** | caaagacagttgactgtatcgccg*gaattc*-ATGTCATCTGGCGGTGTTGCCATT | pBD-EcoRI *– bccdc24* | Cloning – amplification of *bccdc24* for integration in pBD-GAL4 Cam |
| ***Bccdc24-*BD-R** | ttcgcccggaattagcttggctgc*gtcgac*-TTAACGCTCGCCGTCAATGCTTAA | pBD-SalI *– bccdc24* | Cloning – amplification of *bccdc24* for integration in pBD-GAL4 Cam |
| ***Bcfar1*-5F** | gtaacgccagggttttcccagtcacgacg-GACCAAGATTGTTACCTGAAGTCG | pRS426-5F – *bcfar1*-5’ | Cloning – amplification of 5' flank for Δ*bcfar1* construct |
| ***Bcfar1*-5R** | atccacttaacgttactgaaatctccaac-CCGCAGCTAGAAACCCAATCG | *Hph – bcfar1*-5’ | Cloning – amplification of 5' flank for Δ*bcfar1* construct |
| ***Bcfar1*-3F** | ctccttcaatatcatcttctgtctccgac-GCGCGAGGTTAGCGCATTTTG | *PtrpC – bcfar1*-3’ | Cloning – amplification of 3' flank for Δ*bcfar1* construct |
| ***Bcfar1*-3R** | gcggataacaatttcacacaggaaacagc-GCCATCCTTGTGCATTCCCCG | pRS426-3R – *bcfar1*-3’ | Cloning – amplification of 3' flank for Δ*bcfar1* construct |
| ***Bcfar1-*HI-F1** | CCGGAGGTGTGCTAAACTGGAAG | *Bcfar1-*5’ | Diagnostic PCR Δ*bcfar1* – homologous integration at 5' |
| ***Bcfar1-*HI-R1** | GGCTAGAGGGGAGACCGAAGAG | *Bcfar1-*3’ | Diagnostic PCR Δ*bcfar1* – homologous integration at 3' |
| ***Bcfar1-*WT-R1** | CGATCCCGACACCAAGCCGCCAAC | *Bcfar1* ORF | Diagnostic PCR Δ*bcfar1* – detection of *bcfar1* alleles |
| ***BcniiA*-hi5F** | GCGGGGTATGGCAGCATGAGTG | *BcniiA locus* | Diagnostic PCR – homologous integration at *bcniiA*-5' |
| **T*gluc*-hiF** | CATACGTACATCTGATTTGACAACC | T*gluc* | Diagnostic PCR – homologous integration at 5' |
| ***BcniiA*-hi3R** | CTTATAGCAAGCGCGATGTGTATC | *BcniiA locus* | Diagnostic PCR – homologous integration at *bcniiA*-3' |
| ***Nat1*-hiF** | CGGCGAGCAGGCGCTCTACATGAGC | *Nat1* | Diagnostic PCR – homologous integration at 3' |
